# The relationship between procedural volume and patient outcomes for percutaneous coronary interventions: a systematic review and meta-analysis

**DOI:** 10.12688/hrbopenres.13203.1

**Published:** 2021-01-28

**Authors:** Kieran A. Walsh, Thomas Plunkett, Kirsty K. O'Brien, Conor Teljeur, Susan M. Smith, Patricia Harrington, Máirín Ryan

**Affiliations:** 1 Health Technology Assessment (HTA) Directorate, Health Information and Quality Authority, Dublin 7, Ireland; 2 Health Research Board Centre for Primary Care Research, Department of General Practice, Royal College of Surgeons in Ireland, Dublin 2, Ireland; 3 Department of Pharmacology & Therapeutics, Trinity College Dublin, Dublin 8, Ireland

**Keywords:** Systematic review, meta-analysis, volume-outcome, PCI, health services research, STEMI, heart attack, myocardial infarction.

## Abstract

Background: The relationship between procedural volume and outcomes for percutaneous coronary interventions (PCI) is contentious, with previous reviews suggesting an inverse volume-outcome relationship. The aim of this study was to systematically review contemporary evidence to re-examine this relationship.

Methods: A systematic review and meta-analysis was undertaken to examine the relationship between PCI procedural volume (both at hospital- and operator-levels) and outcomes in adults. The primary outcome was mortality. The secondary outcomes were complications, healthcare utilisation and process outcomes. Searches were conducted from 1 January 2008 to 28 May 2019. Certainty of the evidence was assessed using ‘Grading of Recommendations, Assessment, Development and Evaluations’ (GRADE). Screening, data extraction, quality appraisal and GRADE assessments were conducted independently by two reviewers.

Results: Of 1,154 unique records retrieved, 22 observational studies with 6,432,265 patients were included. No significant association was found between total PCI hospital volume and mortality (odds ratio [OR]: 0.84, 95% confidence interval [CI]: 0.69-1.03, *I ^2^* = 86%). A temporal trend from significant to non-significant pooled effect estimates was observed. The pooled effect estimate for mortality was found to be significantly in favour of high-volume operators for total PCI procedures (OR: 0.77, 95% CI: 0.63-0.94, *I ^2^* = 93%), and for high-volume hospitals for primary PCI procedures (OR: 0.77, 95% CI: 0.62-0.94, *I ^2^* = 78%). Overall, GRADE certainty of evidence was ‘very low’. There were mixed findings for secondary outcomes.

Conclusions: A volume-outcome relationship may exist in certain situations, although this relationship appears to be attenuating with time, and there is ‘very low’ certainty of evidence. While volume might be important, it should not be the only standard used to define an acceptable PCI service and a broader evaluation of quality metrics should be considered that encompass patient experience and clinical outcomes.

**Systematic review registration:** PROSPERO, CRD42019125288

## Introduction

The volume-outcome relationship refers to the association between volumes of procedures and outcomes ^1^. The hypothesis underpinning this relationship is that ‘practice makes perfect’, i.e. hospitals or operators that perform a larger number of procedures will achieve better outcomes than those that perform relatively fewer procedures ^2, 3^. Previous systematic reviews have examined the relationship between percutaneous coronary interventions (PCI) volume and postoperative mortality ^4– 6^. A systematic review and meta-analysis published in 2010 investigated the volume-outcome relationship for all PCI procedures, exclusively at the hospital-level ^4^. The authors calculated a pooled effect estimate with an odds ratio (OR) of 0.87 (95% confidence interval [CI]: 0.83-0.91, *I ^2^* = 38%) in favour of high-volume hospitals. A more recent systematic review and meta-analysis published in 2016, also evaluated this relationship at the hospital-level ^5^, again finding in favour of high-volume hospitals (OR: 0.79, 95% CI: 0.72-0.86, *I ^2^* = 38). A systematic review and meta-analysis published in 2014, investigated the volume-outcome relationship at the operator-level ^6^. The pooled effect estimate showed no significant relationship between operator volume and mortality (OR: 0.96, 95% CI: 0.86-1.08, *I ^2^* = 61%) though a significant inverse relationship was found between operator volume and major adverse cardiac events [MACE] (OR: 0.62, 95% CI: 0.40-0.97, *I ^2^* = 97%). 

Evidence from studies in these reviews has informed the development of minimum volume criteria ^7, 8^, which in turn have informed healthcare policy and service provision standards ^9– 11^. However, many have argued that minimum volume criteria should no longer be prioritised as a key metric for PCI service delivery given advances in PCI techniques and postoperative medical management, and regionalisation of care which may have attenuated the volume-outcome relationship over time ^9, 12^. Furthermore, given the inclusion of older, often poorer quality studies in these previous systematic reviews ^4– 6^, an updated review of the volume-outcome relationship at both the hospital- and operator-level is warranted. The aim of this study is to re-examine the relationship between PCI procedural volume and patient outcomes, in light of advances in interventional cardiology and emerging evidence.

## Methods

A systematic review and meta-analysis was undertaken to examine the relationship between procedural volumes (both at the hospital- and operator-level) and patient outcomes, in adults requiring PCI. The primary outcome was mortality. The secondary outcomes were procedural complications, healthcare utilisation and process outcomes. This study is reported according to the Preferred Reporting Items for Systematic Reviews and Meta-Analyses (PRISMA) statement ( *Reporting guidelines* ^13^) ^14^. The protocol was registered on PROSPERO ( CRD42019125288).

### Search strategy

Electronic searches were conducted in PubMed, Embase, CINAHL Plus and the Cochrane Library for the period 1 January 2008 to 28 May 2019. Due to significant advances in PCI practices and perioperative management, only studies examining contemporary evidence, published since 2008 were included ^12^. Grey literature sources were searched ( *Extended data:* S2 ^13^), along with the first five pages of Google and Google Scholar. The search strategy used search terms ( *Extended data:* S3 ^13^) adapted from an earlier systematic review ^5^. Additional search methods included forward citation searching of eligible studies, hand searching relevant journals and systematic reviews and searching reference lists of included studies.

### Study selection criteria, data extraction and quality appraisal

Published observational studies examining the relationship between total PCI (PCI for any acute or elective indication) or primary PCI (PPCI) (emergent angioplasty without the previous administration of fibrinolytic therapy for ST-elevation Myocardial Infarction [STEMI]) procedural volume and patient outcomes were included according to the inclusion and exclusion criteria in Table 1. Screening, data extraction and quality appraisal were all conducted independently by two reviewers from the research team with any disagreements being resolved by discussion, and where necessary, a third reviewer. Covidence ( www.covidence.org) was used for data management and extraction purposes. The data extraction tool was piloted on two studies initially.

**Table 1.** Inclusion and exclusion criteria.

Inclusion Criteria	Exclusion Criteria
■ Relationship between hospital or operator volume and PCI outcomes is investigated PCI defined as follows: ◦ Total PCI - PCI for any acute or elective indication. ◦ Primary PCI - emergent angioplasty without the previous administration of fibrinolytic therapy to open the infarct-related artery during a STEMI. ■ Published observational studies ■ Study uses primary data ■ Study reports at least one of the predefined primary outcomes ■ Study reports adjusted rates ■ For hospital volume studies only: does not describe the results obtained at a single centre ■ For operator studies only: does not describe the results of a single operator	■ Multiple publications based on the same database ■ No definition of procedural volume as a distinct number or cut-off value ■ Conference papers and abstracts where the full paper was unobtainable ■ Paper published prior to 2008 ■ Paediatric (<18 years old) population

PCI – percutaneous coronary intervention; STEMI – ST-elevation myocardial infarction.

The following data were extracted from each included study:

■ year of publication
■ country
■ clinical trial registration
■ database
■ data type (for example administrative or clinical)
■ study period
■ study design
■ number of patients/procedures
■ number of hospitals
■ number of operators
■ hospital volume classification (in terms of cases per year)
■ definition of high-volume hospital
■ definition of high-volume operator
■ how cut-points are selected (for example data-driven, guideline-based)
■ volume grouping (for example quartiles, median)
■ risk adjustment covariates
  — process measures (for example distance to hospital, time to treatment, out of hospital cardiac arrest, radial artery access, use of drug-eluting stents)
  — demographics of patient population (for example sex, indication, stent)
  — patient comorbidities (for example heart failure)
  — hospital cluster effect
  — hospital characteristics (for example presence of on-site surgical cover)
  — severity of disease (for example cardiogenic shock)
  — treatment differences (for example salvage PCI)
■ difference in findings between middle groups and highest/lowest groups.

The primary outcomes:

■ mortality

The secondary outcomes:

■ complications of PCI (for example major adverse cardiac events (MACE)/ major adverse cardiac and cardiovascular events (MACCE), emergency coronary artery bypass graft (CABG), bleeding, peri-procedural myocardial infarction, vascular complications, stroke, contrast-induced nephropathy and stent thrombosis)
■ process outcomes (for example time-to-treatment and appropriateness of PCI)
■ healthcare utilisation outcomes (for example hospital readmission, hospital length of stay, unplanned repeat vascularisations).

A modified version of the Critical Appraisal Skills Programme (CASP) tool for cohort studies was used for quality appraisal at the study-level ^15^. The tool was piloted by two reviewers initially.

### Data synthesis

The methods for data synthesis are summarised here, with greater detail online ( *Extended data:* S4 ^13^). Meta-analysis was performed for the primary outcome, if appropriate to determine the relationship between:

- Hospital volume and postoperative (in-hospital/30-day) mortality
- Operator volume and postoperative (in-hospital/30-day) mortality.

Meta-analyses of adjusted odds ratios (aOR) were conducted separately for studies reporting outcomes for total PCI procedures and for studies reporting outcomes for PPCI procedures only. Pooled estimated effect sizes were calculated using the adjusted outcomes of the highest volume group compared with the lowest volume group. RevMan version 5.3 was used to conduct the random-effects, inverse-variance meta-analysis.

Pre-planned, sensitivity, subgroup and random-effects meta-regression analyses (STATA version 13, StataCorp, College Station, TX, USA) were conducted to explore anticipated heterogeneity and to assess the effect of various studies, subgroups and quality on the overall outcome. Where a temporal trend was observed, a random-effects cumulative meta-analysis was conducted using the median year of study data. A narrative synthesis was undertaken for the findings not included in the meta-analysis.

### Certainty of the evidence

The certainty of the evidence for each primary outcome was assessed independently by two reviewers using the ‘Grading of Recommendations, Assessment, Development and Evaluations’ (GRADE) approach ^16^. A summary of findings table using the GRADEpro software were generated for the primary outcomes ^17^.

## Results

### Search results

**Figure 1.**
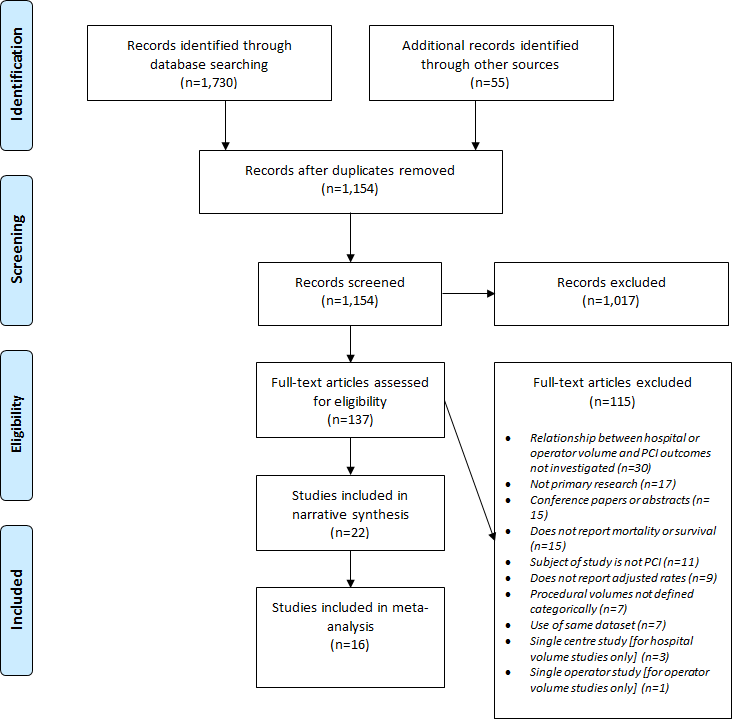
PRISMA Flow Diagram of Included Studies. PRISMA, Preferred Reporting Items for Systematic Reviews and Meta-Analyses; PCI, percutaneous coronary intervention.

Searching electronic databases identified 1,730 records; searching grey literature and other sources identified another 55. After removal of duplicates, 1,154 records were screened, with 1,017 excluded based on titles and abstracts, leaving a total of 137 full-text articles to be assessed for eligibility. Of these, 115 references were excluded ( *Extended data:* S5 ^13^). This resulted in 22 studies being included, of which 16 were included in meta-analyses ( Figure 1).

### Characteristics of included studies

***Study country, population and design.*** Of the 22 included studies, ten were conducted in the US ^18– 27^, with the remainder conducted in Japan (n=5) ^28– 32^, England and Wales (n=2) ^33, 34^, South Korea (n=1) ^35^, Italy (n=1) ^36^, China (n=1) ^37^, Taiwan (n=1) ^38^ and Germany (n=1) ^39^. In total, 6,432,265 patients or procedures were included across the 22 studies. Median or mean age of the study populations ranged from 59 years ^21^ to 74 years ^23^. The majority of included patients were male, ranging from 62% ^23^ to 97.9% ^21^. All included studies were observational; with 20 cross-sectional studies ^18– 20, 22– 36, 38, 39^ and two cohort studies ^21, 37^. Details of the individual study characteristics and outcomes, and the level of analysis of included studies (i.e. hospital-level vs. operator-level) are outlined in the *Extended data* (S6 and S7, respectively) ^13^.

***Definition of high- and low-volume.*** Definitions of high and low volume varied between studies. Some studies developed thresholds that were predominantly data-driven ^19, 20, 26, 29, 30, 32, 36, 39^ (for example, dividing the population into two or more equal sized groups), some developed thresholds that were predominantly guideline-driven ^21– 25, 27, 28, 31, 33– 35, 38^, and others did not provide any clear explanation ^18, 37^.

How the volumes were grouped (tertiles, quartiles, etc) varied substantially between studies, with the number of quantiles ranging from two ^21, 26– 28, 31, 33, 36– 39^ to 10 ^29^. Studies also tested the effect of changing the threshold, or how the threshold/volume was calculated, on the overall outcome ^22, 25– 27, 30, 33, 38, 39^. Some of the alternative thresholds changed the results of the study ^26, 27, 39^.

In general, the proportion of PCI procedures provided in low-volume settings or by low-volume providers decreased over time. When comparing the oldest with the newest studies, the proportion of procedures provided in low-volume settings decreased from: 20.6% ^39^ to 2.1% ^27^ for total PCI and 57.2% ^31^ to 0.07% ^34^ for PPCI; while for low-volume operators the proportion of procedures decreased from 25% ^20^ to 4.8% ^27^ for total PCI. The lowest volume groupings were found to perform a disproportionately higher number of emergent procedures than their higher volume counterparts in six of eight studies that reported this information ^18, 20, 22, 23, 28, 39^.

### Primary outcomes

Postoperative mortality rates (aggregated at the study level) ranged from 0.9% ^29^ to 2.6% ^33^ following total PCI procedures, with a mean mortality rate of 1.5%. For patients undergoing PPCI procedures, postoperative mortality rates ranged from 3.2% ^25^ to 10.1% ^31^, with a mean mortality rate of 5.3% ( *Extended data:* S6 ^13^).

**Figure 2.**
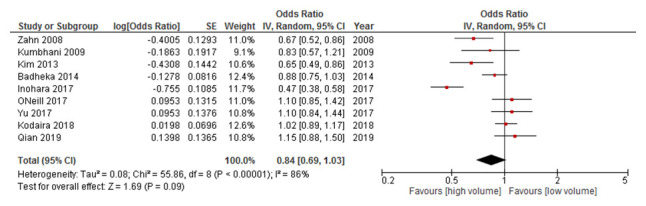
Results of the meta-analysis investigating the relationship between hospital volume and mortality, for total PCI procedures. Studies are arranged in chronolological order based on date of publication. Point estimates to the left of, and confidence intervals not crossing the “line of no effect”, indicate that the effect favours high volume. CI, confidence interval; IV, inverse variance; PCI, percutaneous coronary intervention; SE, standard error.

***Total PCI at the hospital-level.*** For total PCI procedures, the relationship between hospital volume and mortality, was investigated in nine studies ^20, 25, 27– 29, 34, 35, 38, 39^ with no statistically significant difference found between the highest and the lowest volume hospitals (OR: 0.84, 95% CI: 0.69-1.03) ( Figure 2). Of note there was considerable heterogeneity ( *I ^2^* = 86%).

**Figure 3.**
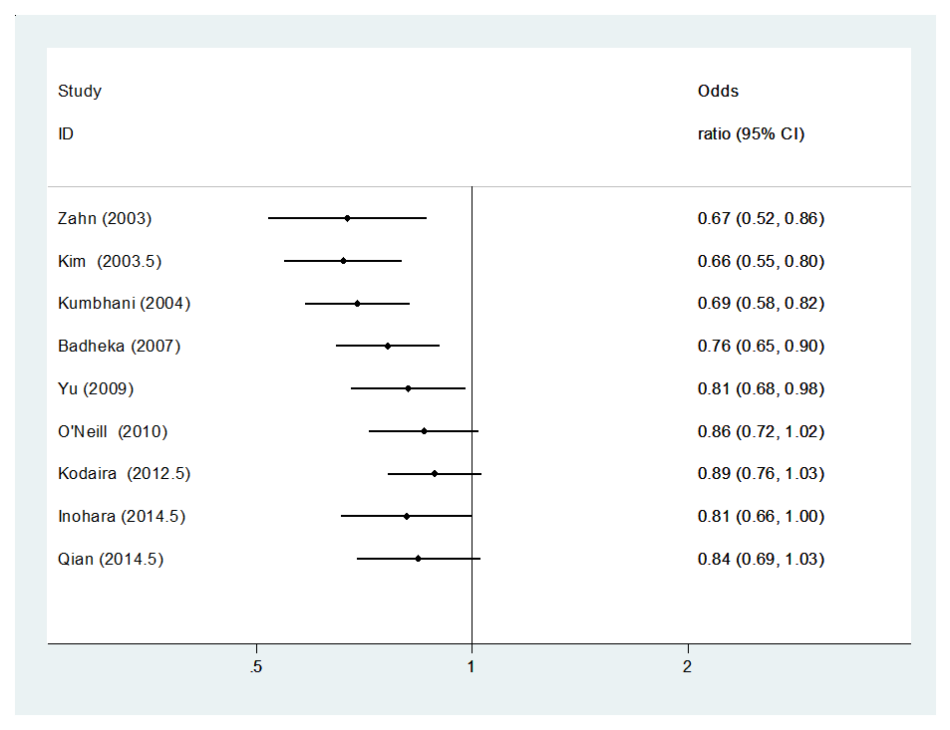
Cumulative meta-analysis investigating the relationship between hospital volume and mortality, for total PCI procedures. Studies are arranged in chronolological order based on median year of data collection. Point estimates to the left of, and confidence intervals not crossing the “line of no effect”, indicate that the effect favours high volume. CI, confidence interval; ID, Identification.

A temporal trend is evident when a cumulative meta-analysis was conducted ( Figure 3). A gradual change can be seen with the pooled effect estimate shifting from a 33% reduction in the odds of mortality (OR: 0.67, 95% CI: 0.52-0.86) when limited to the earliest study data ^39^ to a 16% reduction (OR: 0.84, 95% CI: 0.69-1.03) when data from the most recent studies are included ^27^. Notably, the difference is no longer statistically significant when study data from the year 2010 onwards is included ^34^.

From the exploratory meta-regression analyses, no covariate reached statistical significance ( *Extended data:* S8 ^13^). Subgroup analyses indicated that the overall pooled effect estimate was sensitive to risk of bias and case-mix adjustment. Sensitivity analyses found that the overall pooled effect estimate remains non-significant under all alternative threshold scenarios, except when a threshold of 400 PCI procedures per year or higher was used in one study ^27^, indicating the impact that shifting the threshold can have on the overall findings ( *Extended data:* S9 ^13^).

**Figure 4.**
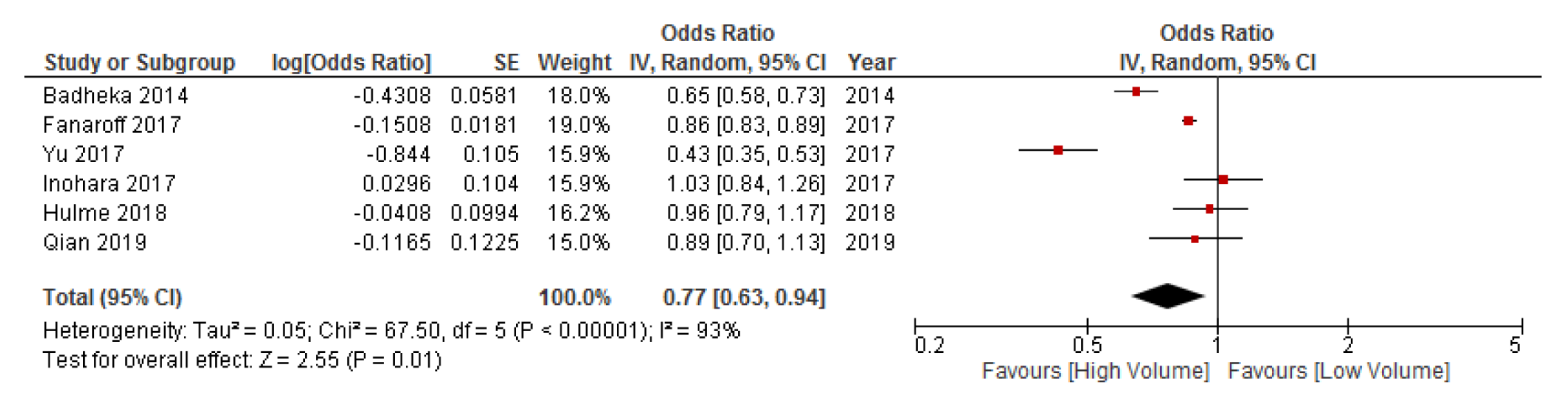
Results of the meta-analysis investigating the relationship between operator volume and mortality, for total PCI procedures. Studies are arranged in chronolological order based on date of publication. Point estimates to the left of, and confidence intervals not crossing the “line of no effect”, indicate that the effect favours high volume. CI, confidence interval; IV, inverse variance; PCI, percutaneous coronary intervention; SE, standard error.

***Total PCI at the operator-level.*** Six studies investigated the relationship between operator volume and mortality, for all PCI procedures ( Figure 4) ^20, 22, 27, 29, 33, 38^. The pooled effect estimate was found to be significantly in favour of high-volume operators (OR: 0.77, 95% CI: 0.63-0.94). Of note there was considerable heterogeneity ( *I ^2^* = 93%).

From the exploratory meta-regression analyses, no covariate reached statistical significance ( *Extended data:* S8 ^13^). Subgroup analyses did not reveal any significant between-group differences. In sensitivity analyses, a study-by-study exclusion process found that the volume-outcome relationship remains significant except when the studies by Badheka *et al.* ^20^ or Fanaroff *et al.* ^22^ ** are removed. Thus, indicating the strong influence of these large population-based studies ( *Extended data:* S9 ^13^).

**Figure 5.**
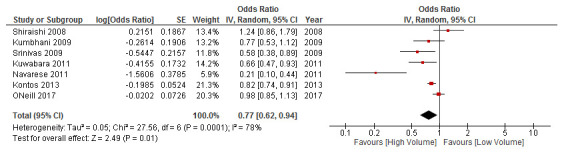
Results of the meta-analysis investigating the relationship between hospital volume and mortality, for primary PCI procedures. Studies are arranged in chronolological order based on date of publication. Point estimates to the left of, and confidence intervals not crossing the “line of no effect”, indicate that the effect favours high volume. CI, confidence interval; IV, inverse variance; PCI, percutaneous coronary intervention; SE, standard error.

***Primary PCI at the hospital-level.*** Seven studies investigated the relationship between hospital volume and mortality, specifically for PPCI procedures ( Figure 5) ^24– 26, 30, 31, 34, 36^. The pooled effect estimate was found to be significantly in favour of high-volume hospitals (OR: 0.77, 95% CI: 0.62-0.94). Heterogeneity again was considerable ( *I ^2^* = 78%).

From the exploratory meta-regression analyses, no covariate reached statistical significance ( *Extended data:* S8 ^13^). Subgroup analyses indicated that the overall pooled effect estimate was sensitive to the mortality outcome used. As only one study in this meta-analysis used 30-day mortality rates ^34^, caution is needed when interpreting this finding. Sensitivity analyses did not reveal any factor that changed the significance of the overall pooled effect estimate ( *Extended data:* S9 ^13^).

***Primary PCI at the operator-level.*** Only two studies investigated the relationship between PPCI at the operator level and mortality, hence a meta-analysis was not conducted ^26, 33^. A 2009 study based on procedures conducted on 7,321 patients between 2000 and 2002, found the odds of mortality were 34% lower (OR: 0.66, 95% CI: 0.48-0.91) for procedures undertaken by operators completing more than 10 PPCI procedures annually compared with those undertaken by operators performing 10 or fewer procedures ^26^. However, a larger (n=133,970) study based on more recent data (2013–2014) found no significant association between operator volume (at a threshold of 75 total PCI procedures per year) and mortality, following PPCI (OR: 0.93, 95% CI: 0.72-1.20) ^33^.

***Minimum volume threshold.*** Ten studies reported annual thresholds above which the adjusted odds ratio for mortality became non-significant ^20, 22, 24, 26, 29, 30, 35, 36, 38, 39^. These cut-points ranged from 208 ^29^ to 400 procedures ^35^ for total PCI hospital volume; from 15 ^20^ to 100 ^22^ for total PCI operator volume; from 36 ^24^ to 66 ^36^ for PPCI hospital volume; and 10 in the sole study which used PPCI operator cut-points ^26^. However, in three studies that had more than two quantiles, some intermediate groups were found to have better outcomes than adjacent higher volume groups ^18, 29, 32^. Six studies conducted spline analysis to investigate the dynamic relationship between volume and outcome ^20, 22, 26, 29, 33, 36^; the estimated optimum annual thresholds varied between studies, ranging from 100 ^29^ to 1,000 ^20^ total PCI hospital procedures.

***Long-term mortality outcomes.*** Two studies investigated the relationship between volume and long-term (i.e., greater than 30 days) primary outcomes ^23, 37^. After adjustment for confounders, no significant difference in mortality between high- and low-volume operators was found at one year (Hazard ratio (HR): 1.04, 95% CI: 1.00-1.08) ^23^ or three years (HR: 0.70, 95% CI: 0.45–1.11) ^37^ post-procedure. Notably, both studies had found significant differences in mortality between high- and low-volume groups at in-hospital ^23^ and 30-day time-points ^23, 37^. 

### Secondary outcomes

A number of secondary outcomes were reported across twelve studies ^19– 25, 27– 29, 32, 37^. These included healthcare utilisation or process outcomes (length of stay ^19, 20^, door-to-balloon [DTB] time ^24, 25^, re-admission ^21^, and inappropriate use of PCI ^27^) and PCI complication outcomes (bleeding ^22, 23, 32^, dialysis ^22^, recurrent MI ^23, 37^, unplanned revascularisations ^23, 37^, and a range of composite outcomes including MACE) ^19, 20, 23, 28, 29, 33, 37^. A consistently significant relationship between procedural volume and healthcare utilisation or process outcomes, was found in favour of high-volume operators and hospitals ^19, 20, 24, 25^, with the exception of PCI procedure appropriateness, which had conflicting findings ^22, 23, 27^. Mixed evidence was also found for the relationship between procedural volume and PCI complications ( *Extended data:* S10 ^13^).

### Quality appraisal

**Figure 6.**
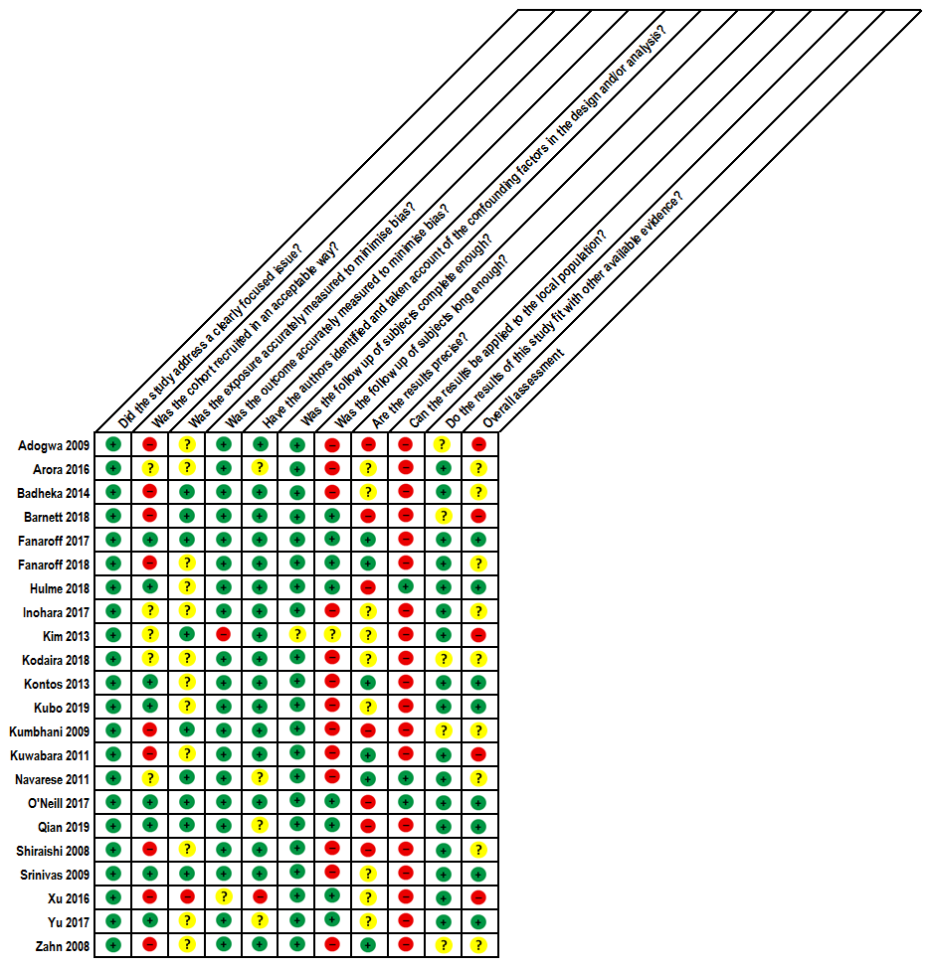
Quality Appraisal of Included Studies using the CASP tool. A modified version of the CASP tool for cohort studies was used for quality appraisal at the study-level. CASP. Critical Appraisal Skills Programme.

Using the CASP quality appraisal tool ^15^, eight studies were judged to have an overall low risk of bias ^22, 24, 26, 27, 32– 34, 38^, nine an unclear risk of bias ^19, 20, 23, 25, 28, 29, 31, 36, 39^ and five a high risk of bias ( Figure 6) ^18, 21, 30, 35, 37^.

### Certainty of the evidence

**Figure 7.**
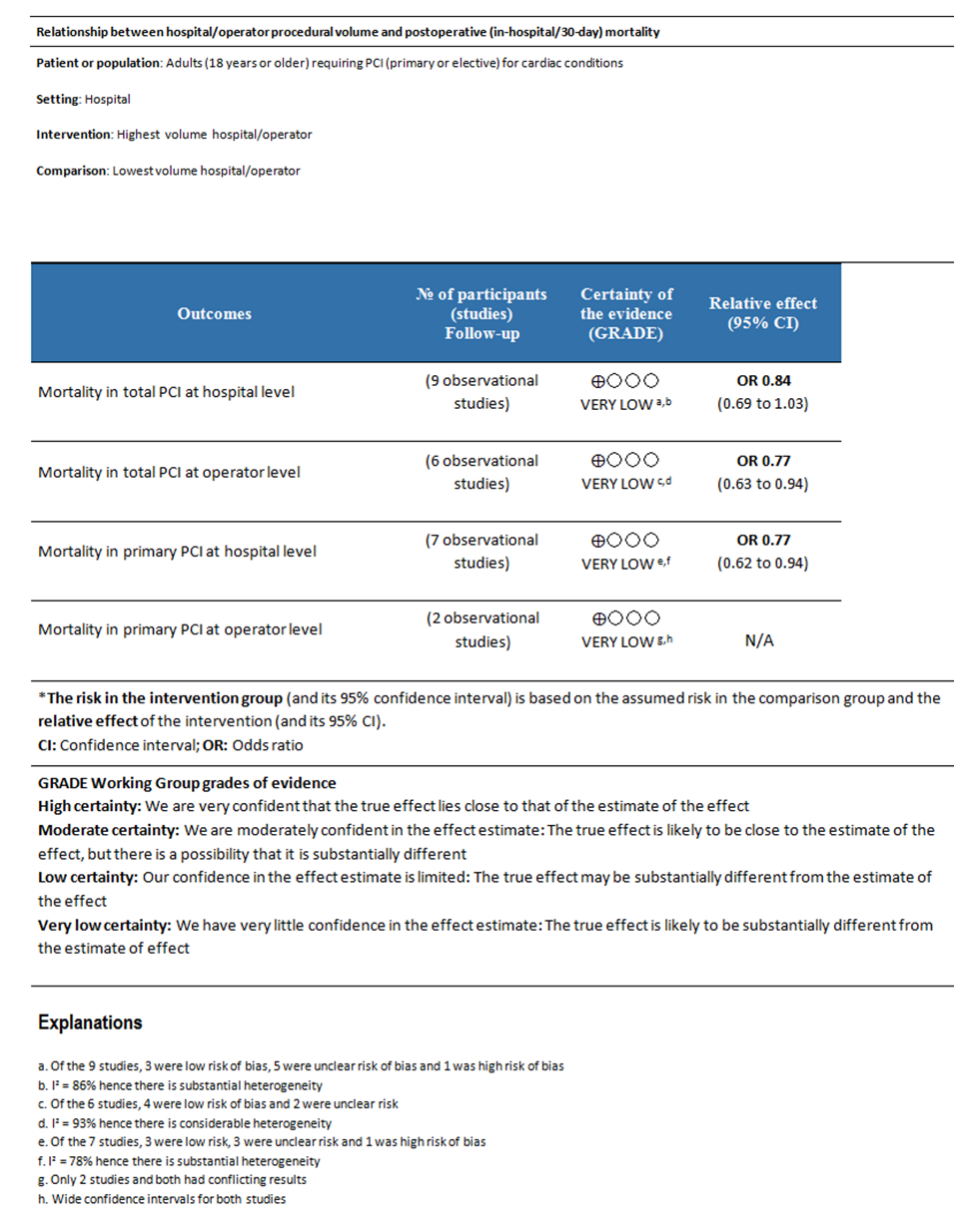
GRADE Summary of findings table. A summary of findings table using the GRADEpro software were generated for the primary outcome. GRADE, Grading of Recommendations, Assessment, Development and Evaluations.

Overall, the certainty of the evidence is ‘very low’ owing to the observational nature of included studies, a high or unclear risk of bias across many included studies, considerable levels of heterogeneity and some concerns regarding the imprecision of results ( Figure 7).

## Discussion

This systematic review and meta-analysis of contemporary research suggests that a significant inverse relationship between PCI procedural volume and patient outcomes may exist in certain situations, however results must be interpreted with caution due to the very high levels of heterogeneity and the ‘very low’ certainty of the evidence. Specifically, no significant association was found between total PCI hospital volume and postoperative mortality, with a temporal trend observed from significant to non-significant pooled effect estimates. Conversely, a significant inverse volume-outcome relationship was found between total PCI operator volume and postoperative mortality, as well as between PPCI hospital volume and postoperative mortality. There is some evidence to suggest that high-volume hospitals offer other benefits in terms of association with reduced length of stay and a greater likelihood of achieving target DTB times. However, there is also evidence to suggest that most, if not all, of the benefits conferred by high-volume operators are attenuated after 30 days ^23, 37^. Due to the huge variability in how studies defined low- and high-volume and differences in how they analysed the data, it is not possible to determine with any degree of certainty, a threshold above which the volume-outcome relationship becomes non-significant.

A key finding of our study was the temporal trend from significant to non-significant pooled effect estimates observed for total PCI hospital volume. With advances in interventional cardiology including increasingly sophisticated operating techniques, more effective drug-eluting stents and improvements in medical management, it is likely that some of these factors may have mitigated the importance of volume on mortality ^12^. Furthermore, introduction of advanced systems of care, streamlined processes and governance structures may also have improved standards across the board ^7^. Due to the implementation of minimum volume standards, it is possible that the observed decrease in the proportion of low-volume hospitals and operators over time may also have moderated the volume-outcome relationship ^34^. What constitutes ‘low-volume’ appears to have changed over time, and the use of traditional cut-points may no longer be sufficiently sensitive to detect a significant difference in outcomes. However, no temporal change was apparent for PPCI hospital volume. Sensitivity analyses conducted for this outcome suggests that there is robustness around this particular pooled effect estimate. Differences in PPCI and total PCI findings may reflect differences in clinical complexity and decision-making in emergent compared with elective cases.

Our findings contrast with those of the earlier systematic reviews ^4– 6^. Our systematic review does however indicate that a significant volume-outcome relationship may exist at the hospital-level for PPCI procedures, and at the operator-level for total PCI. Outcomes from studies evaluating primary and total PCI procedures were combined in previous systematic reviews ^4– 6^, which may have introduced bias into the overall findings due to the inherently higher risk of mortality in STEMI patients and the likely significant confounding due to case-mix in all of these studies. Furthermore, these previous systematic reviews included studies based on PCI data as old as 1984 ^4^, 1990 ^6^ and 1996 ^5^. In contrast, our systematic review which is based on PCI data ranging from 2000 to 2016, provides a more contemporary evidence base.

The main strength of this review was the comprehensive search, in-depth analysis and confirmatory methods including meta-regression, adopted by a team of reviewers experienced in the conduct of systematic reviews. Furthermore, by focusing on more recent data our pooled effect estimates likely better reflect current practice than those calculated by previous systematic reviews ^4– 6^. One of the main limitations of this study was the evidence of a considerable level of heterogeneity in the quality and design of studies; hence the calculated pooled effect estimates must be viewed with caution. Moreover, due to the observational nature of included studies, there are inherent issues surrounding unknown confounding and imbalanced case-mix which may have introduced bias into the results. To address these issues meta-regression, subgroup analyses and sensitivity analyses were conducted. However, no definitive cause of heterogeneity could be determined. Due to the limited number of included studies, the meta-regression analyses should be treated as exploratory and we urge caution in their interpretation. 

Our findings have implications for policy and practice. As health care systems aim to create STEMI networks based on hub-and-spoke models ^40^, our findings suggest there may be a potential mortality benefit of high-volume centres for PPCI procedures in particular. However, an important observation among included studies was that a disproportionately higher number of emergent procedures tended to be conducted by low-volume hospitals and operators ^18, 20, 22, 23, 28, 39^. This finding indicates the important role these low-volume hospitals and operators may have in terms of serving sparsely populated regions. Although enforcing minimum volume standards has been argued as a means of improving standards of care across the board ^34^, it has also been argued that this may unintentionally incentivise some operators and hospitals to perform PCI in patients who have a lower capacity to benefit from the procedure, in order to meet minimum volume requirements ^12^. Furthermore, some have argued that volume in isolation should no longer be prioritised as a key metric for PCI service delivery, and instead a more holistic and multifaceted approach to quality assessment should be adopted ^9, 12^. Therefore, service planners need to carefully balance the need to organise STEMI networks around high-volume centres and operators, while meeting the needs of the entire population, in order to achieve a high-quality, efficient and equitable system with good patient outcomes. Further research is required to inform the optimum configuration of such a network.

In conclusion, this systematic review and meta-analysis suggests a significant inverse relationship between PCI procedural volume and patient outcomes may exist in certain situations; however, results must be interpreted with caution due to the very high levels of heterogeneity and the ‘very low’ certainty of the evidence. A temporal trend was observed, indicating that the relationship between total PCI hospital volume and mortality may be attenuating over time. While a volume-outcome relationship may exist under certain circumstances and might be important, volume should not be the only standard used to define an acceptable PCI service and a broader evaluation of quality metrics should be considered that encompass patient experience and clinical outcomes.

## Data availability

### Underlying data

All data underlying the results are available as part of the article and no additional source data are required.

### Extended data

Figshare: Extended Data: The relationship between procedural volume and patient outcomes for PCI, https://doi.org/10.6084/m9.figshare.13388060.v1 ^13^

This project contains the following extended data:

■ Grey literature sources (S2)
■ Search Terms (S3)
■ Detailed Methods (S4)
■ Studies Excluded After Full Text Review (S5)
■ Characteristics of Included Studies (S6)
■ Level of Analysis in Included Studies (S7)
■ Meta-regression Analyses (S8)
■ Sub-group and Sensitivity Analyses (S9)
■ Secondary Outcomes (S10)

### Reporting guidelines

Figshare. PRISMA checklist for ‘The relationship between procedural volume and patient outcomes for percutaneous coronary interventions: a systematic review and meta-analysis.’, https://doi.org/10.6084/m9.figshare.13388060.v1 ^13^

Data are available under the terms of the Creative Commons Attribution 4.0 International license (CC-BY 4.0).
