# Supplementary material for: The relationship between procedural volume and patient outcomes for percutaneous coronary interventions: a systematic review and meta-analysis
Source: HRB Open Res. 2021 Jan 28;4:10. [Version 1] doi: 10.12688/hrbopenres.13203.1 (PMC8008355; doi:10.12688/hrbopenres.13203.1)
Supplement: Peer review history

# Peer review: Reviewer response for version 1

**DOI:** 10.21956/hrbopenres.14352.r29186

**Published:** 2021-03-26

**Authors:** JJ Coughlan

**Affiliations:** 1 Deutsches Herzzentrum München, Technische Universität München, Munich, Germany

Thank you for inviting me to review this article. The authors are to be congratulated for tackling this challenging topic which may have important implications for care delivery. 

**Are the rationale for, and objectives of, the Systematic Review clearly stated?**

 The rationale behind this systematic review is sound However, with regard to objectives, I felt that these were not as clear. The primary outcome was mortality. I note that from the article, this primarily seems to be in hospital/ 30 day mortality with only a minority of studies reporting on mortality at 1 year. This is an important aspect of this analysis. In hospital/ 30 day Mortality post elective PCI procedures is quite rare and generally estimated to be under 1%. In hospital/30 day mortality post STEMI treated with PPCI is generally higher (~5% depending on the source). 

 I think the question of whether In hospital/ 30 day mortality is affected by volume is perhaps more relevant for ACS or PPCI for STEMI rather than elective PCI. 

 The review would be more useful if the objectives were more clear. Or if, for example the authors specify in their methods the scenarios in which they are going to examine the impact of PCI volume (All PCI, PPCI, ACS, etc), what they are referring to by volume (hospital or operator) and then specify the time frame they are referring to for mortality (it appears to be in hospital/30 day mortality in this study but only one study showed 30 day mortality? It was slightly challenging for me to figure this out when reading the article). 

 The issue with these situations is that a patient may suffer a complication during a PPCI procedure or have delay in opening the infarct related artery, resulting in a significant infarct. They may survive initially but then die from heart failure some time later. 

 I also think that in many ways, operator volume is more relevant than hospital volume and perhaps this should be given primacy when considering the results. 

 So in summary, I think that the authors could perhaps be a bit more specific with regard to their objectives and define more exactly the scenarios in which they are examining this.

**Are sufficient details of the methods and analysis provided to allow replication by others?**

 Yes, the authors have done an excellent job in detailing their methods and analysis which are clear. 

**Is the statistical analysis and its interpretation appropriate?**

 The authors are again to be congratulated for carrying out a high quality analysis. However, a limitation is the significant heterogeneity in the data. 

 Looking at their results in general I would say that overall they seem to indicate that volume does impact mortality. This is significant for operator volume and mortality. For hospital volume for PPCI. It is non significant for overall PPCI and hospital volume but the point estimate is indicating this also favours higher volume. 

 A significant issue here are the definitions of what is considered 'high volume' and this likely contributes to the heterogeneity of the data. 

 I note also that the secondary outcomes were generally in favour of higher volume operators and hospitals. 

**Are the conclusions drawn adequately supported by the results presented in the review?**

 Overall, the authors have a well balanced discussion and their results are expressed thoroughly and cautiously. 

 I think the topic of PCI complexity must also be taken into account when considering changes in patterns over time. For example, a complex CTO procedure may take several hours and carry a far higher risk of complications and mortality than 5 simple PCIs carried out during this time frame. 

 Higher volume centres may also take on more complex cases, which may mask their overall improved outcomes and it is important to make this clear to the reader who may not be familiar with the specifics of PCI. This may also explain the attenuation over time, as lower volume centres became less common and operators begin to take on more complex cases. For example, it is common for cardiologists to now perform multi vessel PCI for patients who are surgical turn downs for CABG. In the past, these patients may have been medically managed. 

 I think that the most important point made by the authors is that volume should not be seen as a marker of quality in isolation and they should emphasise this. 

 For patients with STEMI, organised systems of care and timely opening of the artery are likely more important for the patient than volume per se. 

 Overall though, I congratulate the authors for taking on this challenging topic and for the comprehensive nature of the review and meta-analysis they have performed.

Are the rationale for, and objectives of, the Systematic Review clearly stated?

Partly

Is the statistical analysis and its interpretation appropriate?

Partly

Are sufficient details of the methods and analysis provided to allow replication by others?

Yes

Are the conclusions drawn adequately supported by the results presented in the review?

Partly

Reviewer Expertise:

Interventional Cardiology

I confirm that I have read this submission and believe that I have an appropriate level of expertise to confirm that it is of an acceptable scientific standard.

# Peer review: Reviewer response for version 1

**DOI:** 10.21956/hrbopenres.14352.r28791

**Published:** 2021-02-10

**Authors:** Alexander Fanaroff

**Affiliations:** 1 Cardiovascular Division, Hospital of the University of Pennsylvania, Philadelphia, PA, USA

In this systematic review and meta-analysis of observational studies examining the association between PCI operator and hospital volume and outcomes, Walsh *et al.* found a significant association between operator volume and short-term post-PCI mortality, but no significant association between hospital volume and short-term post-PCI mortality. This systematic review and meta-analysis was obviously a considerable undertaking, the care the authors took in completing a comprehensive and appropriate survey of the literature is apparent. The choices the authors made with respect to study inclusion are reasonable and justified well. The meta-analysis is reported very well, enough to allow replication of the authors methods, and the authors multiple sensitivity analyses and meta-regressions are well-done.

 There have been several meta-analyses of conducted fairly recently (as the authors discuss in their introduction). Interestingly, the results of this meta-analysis contradict the prior results; a 2016 meta-analysis showed a significant inverse association between hospital PCI volume and mortality, and a 2014 meta-analysis showed no significant association between operator PCI volume and mortality. Compared with these prior meta-analyses, the present one has several strengths. First, there have been several analyses of the association between operator volume and mortality, using registry (rather than administrative) data sources that allow for appropriate adjustment for case mix published since 2014. Second, the authors limited their analysis to studies published since 2008 (with resultant limitation of data collection to PCIs performed after 2000). PCI is a rapidly evolving field, and studies of the association between hospital volume and outcomes performed prior to 2008 may not adequately represent the current state of the field.

 The primary limitation of this meta-analysis is heterogeneity between included studies, which is captured in the authors' I ^2^ estimates and, as they note, reduces the certainty of the evidence they've gathered. In the case of the volume-outcome relationship for PCI, heterogeneity would seem to come, a priori, from multiple sources: 1) volume thresholds selected; 2) completeness of adjustment for case mix; 3) regional effects. The authors appropriately did subgroup analyses to look at these subgroups. Notably, they did find a significant difference in their estimate of the association between hospital volume and mortality, with studies with complete case-mix adjustment showing an inverse relationship between hospital PCI volume and mortality, and studies with incomplete case-mix adjustment showing no difference. High and low volume centers take care of different types of patients (in the U.S., for example, high volume centers do more complex PCI and fewer STEMIs, as a proportion of total PCI) and incomplete case mix adjustment (for clinical presentation, but not PCI complexity, for example), may mask true differences in risk-adjusted outcomes between high- and low-volume centers. The likelihood of bias from incomplete case mix adjustment is substantial enough that the authors should consider presenting this data in the main manuscript.

 One of the challenges of the meta-analytic framework is that it forces the authors to consider every study equally without regard to quality or design. In the case of studies of this type, whether a study uses rich clinical data or administrative data is likely to have a large effect on the completeness of case adjustment, which will have a large effect on any observed outcome differences between operator categories, as case mix differs substantially by operator and hospital volume categories. Incomplete case mix adjustment (for indication but not severity of illness or anatomic complexity) may lead to misleading results. The authors may consider a sensitivity analysis examining results from studies using registry versus administrative data, as those using registry data may afford a more complete case mix adjustment.

 The authors' overall assessment of certainty of the evidence is "very low," largely due to heterogeneity. Looking at large, contemporary studies using detailed clinical/registry data, this does seem to be the case for the association between hospital volume and mortality. But looking only at large, highly contemporary analyses of the association between operator volume and outcomes (Inohara ^1^, Fanaroff ^2^, Hulme ^3^), there are fairly narrow and overlapping confidence intervals suggesting a small benefit with a reasonably high level of certainty. This is not consistent with the authors meta-analytic methods, but considering the varying quality of studies in this field, including studies without regard to quality (especially richness of data) may be misleading. 

 Overall, however, when dealing with such substantial heterogeneity, it is important to be circumspect in the conclusions that one draws, and the authors are appropriately circumspect.

Are the rationale for, and objectives of, the Systematic Review clearly stated?

Yes

Is the statistical analysis and its interpretation appropriate?

Yes

Are sufficient details of the methods and analysis provided to allow replication by others?

Yes

Are the conclusions drawn adequately supported by the results presented in the review?

Yes

Reviewer Expertise:

cardiovascular quality and outcomes research

I confirm that I have read this submission and believe that I have an appropriate level of expertise to confirm that it is of an acceptable scientific standard.

## References

[1] : Impact of Institutional and Operator Volume on Short-Term Outcomes of Percutaneous Coronary Intervention. *JACC: Cardiovascular Interventions*.2017;10(9) : 10.1016/j.jcin.2017.02.015 918-927 10.1016/j.jcin.2017.02.015 28473114

[2] : Outcomes of PCI in Relation to Procedural Characteristics and Operator Volumes in the United States. *J Am Coll Cardiol*.2017;69(24) : 10.1016/j.jacc.2017.04.032 2913-2924 10.1016/j.jacc.2017.04.032 28619191PMC5784411

[3] : Operator volume is not associated with mortality following percutaneous coronary intervention: insights from the British Cardiovascular Intervention Society registry. *European Heart Journal*.2018;39(18) : 10.1093/eurheartj/ehy125 1623-1634 10.1093/eurheartj/ehy125 29579219
